# Physical Activity Perceptions and Participation of People With Type II Diabetes Mellitus in the Dominican Republic

**DOI:** 10.7759/cureus.62608

**Published:** 2024-06-18

**Authors:** Amerigo Rossi, Mónica O Rossi, Camille Palarpalar, Lorenza Almonte, Alex Rothstein, Lillian B Niwagaba

**Affiliations:** 1 Interdisciplinary Health Sciences, New York Institute of Technology, Old Westbury, USA; 2 School of Public Health, City University of New York, New York City, USA; 3 Osteopathic Medicine, New York Institute of Technology, Old Westbury, USA; 4 Nagua Chapter, Rotary International, Inc., Nagua, DOM; 5 Interdisciplinary Health Sciences, New York Institute of Medicine, Old Westbury, USA; 6 Global Health, New York Institute of Technology, Old Westbury, USA

**Keywords:** low- and middle-income country (lmic), diabetes type 2, adjunctive therapies, exercise-based therapy, accelerometer, physical activity level, exercise, godin leisure-time exercise questionnaire, developing countries

## Abstract

Introduction: Physical activity (PA) improves health outcomes for people with type II diabetes mellitus (diabetes), but little is known about PA among Dominicans. The purpose was to evaluate PA participation and perceptions among people with diabetes in the Dominican Republic (DR).

Methods: Participants (N=29) were recruited from an urban diabetes clinic in DR. PA was assessed via accelerometry and Godin Leisure Time Exercise Questionnaire (GLTEQ).

Results: Eighteen women and 11 men enrolled (age: 55 ± 13 years; BMI: 28.6 ± 4.5 kg·m^-2^). Twenty-seven participants reached acceptable wear time. Using a one-minute bout minimum, moderate- to vigorous-intensity PA (MVPA) was 152.2 ± 59.7 min·day^-1^; no vigorous PA was recorded. GLTEQ scores (103 ± 98) classified 25 participants as active. Around 93% reported that PA was “very important” for their health. There was no association between GLTEQ and MVPA (p>0.2). Participants who reported being “very active” (n=17) did more MVPA than those who were “rarely active” or “somewhat active” (n=10; p=0.02).

Conclusion: Dominicans with diabetes are highly physically active but do very little vigorous PA. The GLTEQ was not an accurate measure of PA. Future research should develop validated questionnaires and evaluate structured exercise and dietary interventions.

## Introduction

Type II diabetes mellitus (diabetes) is a chronic illness that affects the body’s ability to process blood glucose, leading to significantly higher morbidity risk and premature mortality [1]. The global burden of diabetes is increasing, with over 6% of the world population now affected [2]. Although most cases occur in high-income nations, there has been a recent alarming increase in low- to middle-income nations [3,4]. For example, in the Dominican Republic (DR), a middle-income country with a population of 11.4 million, diabetes prevalence has increased nearly 25% in the past 10 years, up to a total of 12.7% among adults [5]. The primary challenges to robust diabetes management in the DR have been a lack of health insurance and expensive medications [6].

Despite the growing societal burden of diabetes in the DR, very little research has been conducted to evaluate programs and initiatives to reduce its negative impact. One reason for this lack of research may be a lack of diabetes knowledge among Dominicans. Only 51% of rural Dominicans had ever heard of diabetes [7], and only 28% of people with diabetes had been diagnosed [7], compared to 50% globally [8], and 77% in the United States [8]. Out of 20 Latin American countries, Dominican physicians are the least informed about “Exercise is Medicine” [9].

A program evaluation of two rural diabetes clinics in the DR found that focusing on medication adherence and healthy eating led to a 0.1% reduction in glycated hemoglobin (HbA1c) per year [6], but did not evaluate physical activity (PA), although PA is a powerful complementary component of diabetes management [10]. Only two studies have provided information regarding PA among Dominicans. In one study, 65% of Dominicans experiencing normal aging considered themselves to be “very physically active” or “fairly physically active” [11]. In another, female Dominican college students self-reported 65 minutes per week of PA, which is far below the minimum recommended guidelines to maintain health [12].

Given that over 80% of Dominicans live in urban areas [13] and the heavy societal and economic burden caused by the increase in diabetes prevalence [14], it is vital that we develop evidence-based interventions, including PA programs and education-based interventions, to create sustainable diabetes care centers in urban areas to prevent and treat diabetes. The first step toward that goal should be to develop baseline data regarding PA among Dominicans with diabetes. Therefore, the aim of this study was to be the first to evaluate PA participation and perceptions among people with diabetes in DR.

Portions of this article were previously presented as a meeting abstract at the Spring 2024 American College of Sports Medicine Greater New York, NY, on April 20, 2024.

## Materials and methods

This cross-sectional study was approved by the New York Institute of Technology Institutional Review Board in the United States (BHS-1873), and by the Consejo Nacional de Bioética en Salud (CONABIOS) in the Dominican Republic (043-2023).

**Figure 1:**
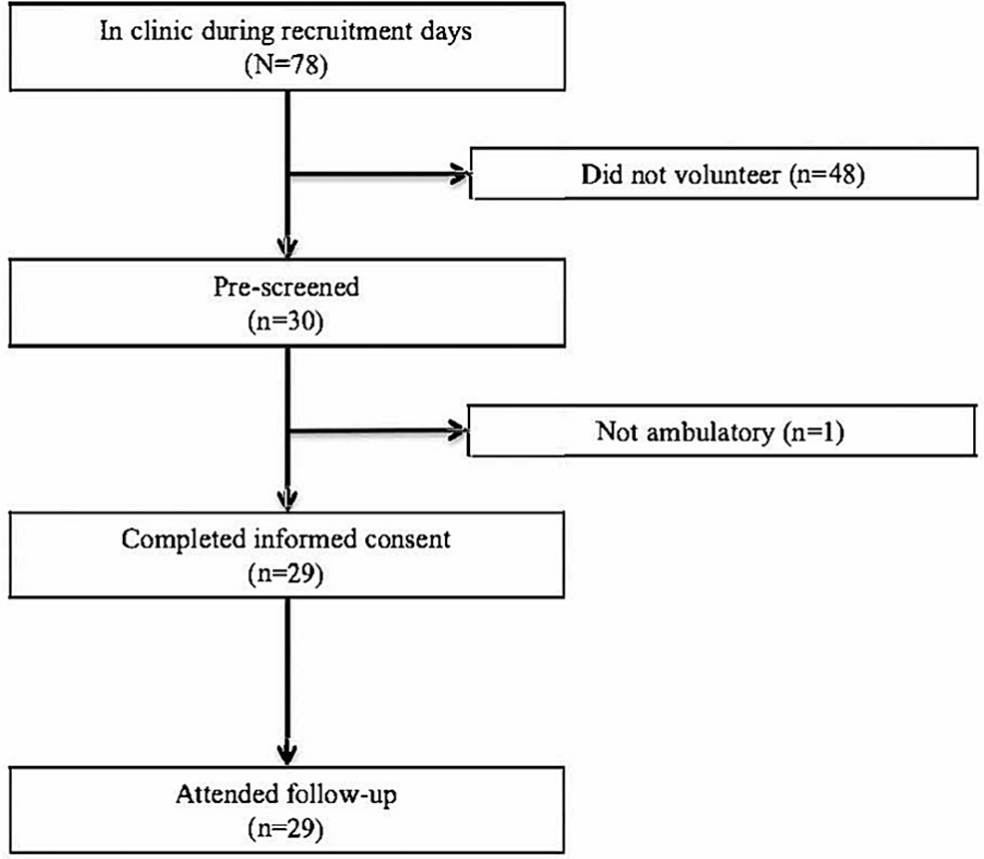
Participant recruitment flow chart

Participants were recruited from an urban diabetes clinic in Nagua, DR, a city of approximately 40,000, during the summer of 2023. Inclusion criteria were a current patient at the Centro Regional Contra la Diabetes diagnosed with diabetes, at least 18 years old, independently ambulatory, and able to communicate in Spanish. Participants were excluded if they were pregnant or unable to return to the center in one week for follow-up testing. Recruitment consisted of posting flyers in the waiting room, and a verbal invitation during each recruitment day from the site administrator. Of the 78 people with diabetes who attended the clinic on recruitment days, 30 expressed interests, and 29 were enrolled in the study (37% recruitment rate) (Figure 1). Reasons for not volunteering were not formally collected but many potential participants said it would be too difficult to return to the clinic the following week. This pilot study originally aimed to recruit 80 participants. However, recruitment time was limited due to challenges in gaining approval from institutional and national ethical review panels.

Eligible participants completed the informed consent process and all testing in a private room. Demographic information was collected via a questionnaire. Height and weight were self-reported. However, the majority of participants did not know their height. In those cases, height was measured using a wall-mounted measuring tape. Participants were then fitted for an accelerometer (Actigraph wGT3X-BT, Pensacola, FL) on their non-dominant wrist. They were instructed to wear the accelerometer as much as possible during the subsequent seven days, except when showering or swimming, and not change their normal routine.

After seven days, participants were to come back to the clinic for a follow-up appointment. During this appointment, they were asked two PA perception questions with Likert-scale responses: “How physically active do you think you are? Never active; Rarely active; Sometimes active; Very active,” and, “How important do you think physical activity is for your health? Not important at all; Slightly important; Moderately important; Very important.” The study personnel also asked them the questions to the Spanish-language Godin Leisure Time Exercise Questionnaire (GLTEQ) [15]. The GLTEQ was selected because it is 1) very simple to complete with just three questions and 2) widely used to evaluate PA in clinical settings [15]. All questionnaires were completed via directed questions because many participants were not fully literate.

Statistical analysis

Actilife software v6.13.6 (ActiGraph, Pensacola, FL, USA) was used to evaluate the PA data. Troiano et al. [16] counts/minute cut points were used to differentiate between light intensity (100-2,019), moderate intensity (2,020-5,998), and vigorous intensity (≥5,999). The Troiano cut points were used because they provide a relatively high threshold of moderate- and vigorous-intensity PA which makes these data fairly conservative [17]. Data were recorded at 30 Hz, and 60-second epochs were used. Non-wear time was defined as bouts of at least one hour without any counts. Accelerometer data were considered acceptable if wear time was ≥10 hours/day on ≥ 4 days [18]. Best practices regarding minimum bout time to accurately capture moderate- to vigorous-intensity PA (MVPA) have not been established. Since this is a preliminary study that may be used as a reference for future research, we used the two widely used bout lengths: one minute [19] and 10 minutes [20] to maximize the generalizability of the findings. The one-minute minimum bout lengths captured short-duration bouts lasting at least one minute, whereas the 10-minute bout lengths only captured sustained PA lasting at least 10 minutes.

Statistical Package for the Social Sciences (IBM Corp., Released 2023. IBM SPSS Statistics for Windows, Version 29.0.2.0; IBM Corp., Armonk, NY) was used for descriptive and inferential statistics. Means and standard deviations were calculated for age and BMI. Chi-squared tests were used to compare frequencies between men and women. Independent samples t-tests with Levene’s test for equality of variances were used to evaluate differences between men and women for age, BMI, MVPA, and GLTEQ scores, as well as for differences in MVPA scores based on participant perceptions. Levene’s test was significant for the GLTEQ (p = 0.001), so the t-test was analyzed without the assumption of equal variances. Pearson’s correlation coefficients were calculated to evaluate the association between MVPA/day, steps/day, and GLTEQ scores. Detection of possible outliers was conducted by calculating the interquartile range (IQR) for outcome variables and setting the cut-offs as greater than Q3 + 1.5IQR or less than Q1-1.5IQR. No outliers were detected. P-value was set a priori at p ≤ 0.05.

## Results

Although many participants lived up to an hour's drive from the clinic and required travel assistance, all 29 participants returned for the follow-up session. Acceptable wear time (≥10 hours/day on ≥4 days) was achieved by 27 participants (93%). The mean participant age was 54.7 ± 13.0 years. Nine participants were 32-49 years old, nine were 50-59 years old, seven were 60-69 years old, and four were 70-80. According to BMI classifications, five participants were healthy weight, 15 were overweight, and nine were obese. All participants were of Dominican descent. There were no differences between genders for demographic data (Table 1).

**Table 1:** Demographic characteristics of participants (mean ± SD or frequency (%)) P-value calculated for differences between men and women

Variable	Total (N=29)	Women (N=18)	Men (N=11)	P-value
Age (years)	54.7 ± 13.0	54.2 ± 14.9	55.5 ± 9.6	0.79
BMI (kg/m^2^)	28.6 ± 4.5	28.6 ± 5.0	28.6 ± 3.9	0.98
Dominican Nationality	29 (100%)	18 (100%)	11 (100%)	N/A

Using the one-minute bout minimum, participants completed 152.2 ± 59.7 minutes of MVPA per day (Table 2). Using the 10-minute bout minimum, MVPA was 34.9 ± 36.0 minutes per day. There were zero bouts of vigorous-intensity PA in either analysis. The mean steps per day were 9,851 ± 3,086, and the Godin Leisure Time Index was 103 ± 97.8, with 25 participants classified as “Active,” two as “Moderately Active,” and two as “Sedentary.” Although the data were not formally captured, many respondents indicated that nearly all of their reported activity came from household chores or yard work. Men reported much higher scores on the GLTEQ (144 vs. 78), but the difference was not significant (p=0.15).

**Table 2:** Physical activity perceptions and outcomes (mean ± SD or frequency (%)) P-value calculated for differences between men and women

Physical activity perception/participation	Total (N=29)	Women (N=18)	Men (N=11)	P-value
How physically active are you?				
Never	0	0	0	0.42
Rarely	3 (10%)	2 (11%)	1 (9%)	
Sometimes	9 (31%)	4 (22%)	5 (45%)	
Very	17 (59%)	12 (67%)	5 (45%)	
How important is physical activity for your health?				
Not at all	0	0	0	0.25
A little	0	0	0	
Somewhat	2 (7%)	2 (11%)	0	
Very	27 (93%)	16 (89%)	11 (100%)	
Godin Leisure Time Exercise Questionnaire Index	103.2 ± 97.8	78.3 ± 59.4	144.0 ± 133.4	0.15
Moderate Intensity Physical Activity, >1-minute bouts (minutes/day)	152.2 ± 59.7	152.7 ± 67.9	151.5 ± 48.6	0.96
Moderate Intensity Physical Activity, >10-minute bouts (minutes/day)	34.9 ± 36.0	42.6 ± 38.5	23.9 ± 30.3	0.19
Steps per day	9,851 ± 3,086	9,633 ± 3,103	10,518 ± 2,861	0.46

Seventeen participants (59%) classified themselves as “very active,” nine (31%) as “sometimes active” and three (10%) as “rarely active.” In order to evaluate differences in PA based on PA perceptions, the “sometimes active” and “rarely active” groups were combined. MVPA minutes per day (166.7 ± 506 vs. 117.8 ± 45.8, p=0.019) and steps per day (10,902 ± 2,702 vs. 8,067 ± 2,983, p=0.018) were significantly higher in the self-reported “very active” group compared to the pooled “sometimes active” and “rarely active” groups.

Out of the 29 participants, 27 (93%) indicated that PA was “very important” to their health, and two (7%) indicated that it was “somewhat important.” There were no significant correlations (p>0.2) between the GLTEQ and either MVPA one-min (r=0.05), MVPA 10-min (r=-0.13), or steps/day (r=0.15) (Table 3). MVPA one-min, MVPA 10-min, and steps/day were all significantly correlated (p<0.05). A follow-up analysis revelaed no significant correlations between GLTEQ question two regarding moderate-intensity PA and either MVPA or steps/day (p>0.20).

**Table 3:** Pearson correlation coefficients (r) between physical activity variables (n=27) *p < 0.05 GLTEQ: Godin Leisure Time Exercise Questionnaire, MVPA: Moderate- to vigorous-intensity physical activity

Physical activity variable	GLTEQ Index	MVPA (≥ 1 min)	MVPA (≥ 10 min)	Steps/day
GLTEQ Index	--	0.05	-0.13	0.15
MVPA (≥ 1 min)		--	0.86*	0.87*
MVPA (≥ 10 min)			--	0.71*

## Discussion

The primary finding of this study was that Dominicans with type II diabetes were highly physically active, but did not engage in vigorous PA. Additionally, the GLTEQ, which has been validated in several populations [21], was not correlated with any objective measure of MVPA in this sample. Lastly, although very little research has been done regarding PA among Dominicans, awareness of the importance of PA for health was high.

The American College of Sports Medicine recommends that people with type II diabetes complete at least 150-300 minutes per week of moderate-intensity PA [22]. The participants in this study completed 1,065 minutes per week, on average, when computing MVPA bouts lasting at least one minute. When using a more conservative model to only include bouts lasting at least 10 minutes, the average MVPA was 244 minutes/week, with 16 out of 27 being classified as sufficiently physically active (>150 min/week). These data contrast with findings from previous studies of people with diabetes from high-income countries using wrist-worn accelerometers that have found approximately 560 minutes per week of MVPA using one-minute bouts, and 52 minutes per week using the more conservative method of 10-minute bouts [23]. The participants in this study were two to five times more physically active, depending on the method of interpretation. In a study of 1,232 adults from the Latin American cities of Bogota, Cuernavaca, and Curitiba, 54%-79% of participants met the recommended volume of PA when using the one-minute bout threshold, and 13%-29% when using the 10-minute threshold [24]. In comparison, 100% of the sample in this study met the recommendations when using the one-minute threshold, and 57% when using the 10-minute threshold. More research with larger samples needs to be completed to confirm these findings.

It is important to note that there were zero detected bouts of vigorous-intensity PA lasting at least one minute, and anecdotal evidence suggests that participants got nearly all of their PA from manual labor in/around their homes instead of from structured physical activities such as walking or exercise classes. These data indicate that, although Dominicans with type II diabetes are physically active, they may benefit from structured PA interventions to introduce resistance training and other vigorous modalities, which may lead to significant improvements in health and wellness in people with diabetes [22].

To the best of our knowledge, this is the first study to objectively evaluate PA among Dominicans. One study, which asked Dominican adults to self-report their PA level, found that 65% of Dominicans experiencing normal aging considered themselves to be “very physically active” or “fairly physically active” [11]. The current study found higher levels of self-reported PA, with 90% considering themselves very physically active (59%) or sometimes active (31%). Another study of Dominicans, which evaluated PA via an adapted short International Physical Activity Questionnaire, found that 16 female college students completed 65 minutes per week of PA [25], which is much lower than what was found in this study via accelerometry. It is likely that objective PA monitoring captures moderate-intensity PA during behaviors that Dominicans may not consider PA, such as brisk walking, household chores, or yard work. Further research validating detailed PA surveys, such as the Yale Physical Activity Survey [26], should be used to develop an informative PA questionnaire for Dominicans.

Because type II diabetes is largely caused by lifestyle factors such as PA and nutrition [27], and these participants were highly physically active, it is reasonable to conceive that nutritional interventions may be particularly impactful in improving disease progression in this population. A recent systematic review indicated that nutrition education alone may improve HbA1c by -0.63% [28] and reduce the incidence of diabetes risk by 30% in low- to middle-income countries [29], although the long-term outcomes remain unclear. Future research should be conducted to develop sustainable community-based nutritional interventions for people with diabetes in the DR to improve disease outcomes. For example, an integrated community garden and peer nutritional counseling program for people living with HIV in DR was found to be feasible and impactful [30] and may serve as a template for targeted nutrition programming development.

One limitation of this study was the small sample size from a single diabetes center. It is possible that these participants are not representative of urban Dominicans with diabetes, such as those from the capital city, Santo Domingo. Follow-up studies should include a larger sample that includes people with diabetes from the capital city as well as rural areas. Furthermore, it is possible that participants who were already knowledgeable about PA chose to participate in the study, or that they increased their PA levels during the course of the study, thereby inflating the measured PA values. We attempted to minimize these risks by offering remuneration to entice those not already interested in PA, and by instructing participants not to change their routine. Additionally, the levels of PA were so much higher than expected that it would be difficult to disregard the magnitude of these findings. Although accelerometry is considered the gold standard for field-based PA assessment, there are several limitations associated with its use, including missing data during times of non-use.

The lack of consistency between the objective and subjective measures of PA made it difficult to triangulate the data, which would have added additional credence to the validity of our findings. The GLTEQ may not have been detailed enough to capture the intricacies of PA in this sample. Future research should administer more detailed PA questionnaires that can also be applied to non-literate individuals.

Height and weight were self-reported to minimize participant burden, which may have introduced some bias in the BMI calculations. However, most participants did not know their height, so they were measured using a wall-mounted measuring tape. Most participants had recently been weighed during their normal clinic visits, so they were aware of their accurate weight. Future studies should include low-burden measurements of height and weight to ensure the accuracy of BMI when that is a primary outcome.

## Conclusions

This was the first study to objectively evaluate the PA of Dominicans. Dominicans with type II diabetes are highly physically active but may benefit from interventions to improve nutrition and structured PA such as resistance training. Apparent differences in self-reported PA between men and women warrant further study with larger sample sizes and may indicate the need for differential PA programming. The subjective assessment of PA was not correlated to objective PA assessment, so more testing should be done to validate low-burden PA questionnaires in this population.

Although previous studies had found diabetes literacy to be very low in this population, this study found that the vast majority of participants that PA was very important for their health, which suggests the opportunity for high adherence to interventions. Future studies should test sustainable nutrition and exercise interventions to improve diabetes outcomes and should test the validity and reliability of culturally adapted PA questionnaires.
